# Molecular transport through primary human small intestinal monolayers by culture on a collagen scaffold with a gradient of chemical cross-linking

**DOI:** 10.1186/s13036-019-0165-4

**Published:** 2019-04-27

**Authors:** Jennifer E. Speer, Dulan B. Gunasekara, Yuli Wang, John K. Fallon, Peter J. Attayek, Philip C. Smith, Christopher E. Sims, Nancy L. Allbritton

**Affiliations:** 1 Department of Chemistry, University of North Carolina, Chapel Hill, NC 27599 USA; 2 Joint Department of Biomedical Engineering, University of North Carolina and North Carolina State University, Raleigh, NC 27599 USA; 3 Division of Pharmacoengineering and Molecular Pharmaceutics, Eshelman School of Pharmacy, University of North Carolina, Chapel Hill, NC 27599 USA

**Keywords:** Intestinal transport, Stiffness, Extracellular matrix, Small intestine

## Abstract

**Background:**

The luminal surface of the small intestine is composed of a monolayer of cells overlying a *lamina propria* comprised of extracellular matrix (ECM) proteins. The ECM provides a porous substrate critical for nutrient exchange and cellular adhesion. The enterocytes within the epithelial monolayer possess proteins such as transporters, carriers, pumps and channels that participate in the movement of drugs, metabolites, ions and amino acids and whose function can be regulated or altered by the properties of the ECM. Here, we characterized expression and function of proteins involved in transport across the human small intestinal epithelium grown on two different culture platforms. One strategy employs a conventional scaffolding method comprised of a thin ECM film overlaying a porous membrane while the other utilizes a thick ECM hydrogel placed on a porous membrane. The thick hydrogel possesses a gradient of chemical cross-linking along its length to provide a softer substrate than that of the ECM film-coated membrane while maintaining mechanical stability.

**Results:**

The monolayers on both platforms possessed goblet cells and abundant enterocytes and were impermeable to Lucifer yellow and fluorescein-dextran (70 kD) indicating high barrier integrity. Multiple transporter proteins were present in both primary-cell culture formats at levels similar to those present in freshly isolated crypts/villi; however, expression of breast cancer resistance protein (BCRP) and multidrug resistance protein 2 (MRP2) in the monolayers on the conventional scaffold was substantially less than that on the gradient cross-linked scaffold and freshly isolated crypts/villi. Monolayers on the conventional scaffold failed to transport the BCRP substrate prazosin while cells on the gradient cross-linked scaffold successfully transported this drug to better mimic the properties of in vivo small intestine.

**Conclusions:**

The results of this comparison highlight the need to create in vitro intestinal transport platforms whose characteristics mimic the *in vivo lamina propria* in order to accurately recapitulate epithelial function.

**Graphical abstract:**

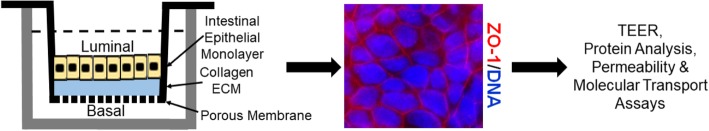

**Electronic supplementary material:**

The online version of this article (10.1186/s13036-019-0165-4) contains supplementary material, which is available to authorized users.

## Background

The luminal surface of the small intestine is composed of a single layer of polarized columnar epithelial cells with the most prevalent cell type being enterocytes, absorptive cells that transport nutrients, salt, metabolites and drugs [1]. The presence of villi lining the intestinal surface greatly increases the absorptive area, while microvilli covering the surface of the enterocytes, known as the brush border, increases the cellular surface area for transport and passive absorption [2, 3]. Proteins expressed by the epithelium facilitate the transport of drugs, metabolites, ions and amino acids [4]. The two major families of drug transporter proteins found in the small intestine are the ATP-binding cassette (ABC) superfamily and the solute carrier (SLC) superfamily, both of which mediate drug and metabolite uptake and export. ABC transporters harness energy from ATP hydrolysis to actively transport substrates across cellular membranes, whereas SLC transporters are primarily involved in the passive uptake of small molecules into cells [5]. The ABC transporter family includes clinically important proteins such as P-glycoprotein (P-gp), multidrug resistance proteins (MRP1, 2, 3), and breast cancer resistance protein (BCRP) [6]. The SLC transporters participate in both absorption and secretion of anionic and cationic molecules and include proteins such as the organic cation transporter 3 (OCT3) [7, 8]. Na^+^/K^+^-*ATPase* and gamma-glutamyl transpeptidase (GGTP) are not part of the two major superfamilies but play important roles in the transport of molecules. The Na^+^/K^+^-*ATPase* enzyme present on the basal aspect of the epithelial cells actively exports sodium while importing potassium, both against their concentration gradient. The resulting Na^+^ gradient drives a Na^+^-glucose symporter on the cells’ luminal face that imports both Na^+^ and glucose in an efficient manner [4, 9]. GGTP is a transferase in the brush border that catalyzes the transfer of gamma-glutamyl functional groups from glutathione to an acceptor such as a peptide or amino acid to form glutamate, and in the intestine is involved in amino acid absorption [10, 11]. To study the function of these various proteins, particularly for drug and nutrient transport, development of a primary, human intestinal epithelial monolayer system providing access to the luminal and basal aspects of the epithelium is a necessary tool.

To perform transport studies, typically human cancer lines, such as Caco-2, are used as a model system as they are inexpensive, easy to culture, are readily available, and can be differentiated to an enterocyte-like cell. While tumor model systems have played a valuable role in drug discovery, these cells often fail to predict in vivo behavior due to inappropriate expression levels or mutations of transporters and enzymes in comparison with the normal intestine [12]. Animal models are also often used to predict intestinal absorption, but are technically difficult, expensive, and face increasing ethical challenges [13]. These non-human model systems may also display levels and physical locations of proteins distinct from that of humans, resulting in inaccurate clinical predictions [14]. Human intestine explant cultures have been used for drug absorption studies, but the short survival times of these systems ex vivo makes their routine use impractical for intestinal transport studies [15]. Enteroids derived from mouse and human intestinal stem cells recapitulate many features of small intestine epithelial cell function. However, enteroids are closed spherical structures embedded within a hydrogel with lumen not easily accessed, thus making the system challenging for use in transport studies [13].

Differentiated intestinal epithelial monolayers derived from primary intestinal stem cells demonstrate much of the in vivo cell architecture and function including proper luminal-to-basal cell polarity, the presence of tight junctions, and all differentiated cell types of the in vivo epithelium [15–18]. When cultured on a porous membrane, such as a Transwell™, these monolayer systems provide ready access to the luminal and basal reservoirs bathing the tissue and enable fluid sampling for influx and efflux studies. To address the need for improved systems for drug assays, a variety of different formats to form epithelial monolayers have been described [16–20]. These systems are most distinguished by the culture surface used, often a thin coating of extracellular matrix (ECM) on a polymer surface such as polycarbonate, polystyrene, or polydimethylsiloxane (PDMS). While these systems demonstrate gene expression at the mRNA level of known transporters, these non-natural polymer culture surfaces can have a profound impact of transporter activity since the underlying stiff surfaces do not replicate most features of the *lamina propria* [21, 22]. Thick collagen scaffolds mimicking critical features of the *lamina propria,* such as its stiffness, have been shown to support both human and mouse primary intestinal monolayers for short-term assay or long-term culture [23–25]. A recently described scaffolding utilizes a gradient of chemical cross-linking across a thick collagen hydrogel so that the luminal collagen surface in contact with cells mimics the stiffness of in vivo intestine while the more cross-linked or rigid basal surface prevents gross hydrogel deformation by the cells [25]. However, the cells on this newer physiologic scaffold have not been characterized with regard to their transporter protein expression and function.

The stiffness of the matrix, such as the *lamina propria*, underlying cells in vivo and in culture has long been known to have a profound influence on cellular physiology, impacting properties as diverse as proliferation, differentiation, protein expression and signal transduction among others [26–28]. For example, ECM stiffness is known to influence stem-cell fate through regulated mechano-sensing pathways [29–31]. Despite a growing interest, limited studies of the interplay of stiffness and stem cell self-renewal, cellular differentiation and cell function have been performed in intestinal epithelium. Organoid culture systems have shown optimal intestinal cell growth in stiff matrices and optimal differentiation in soft matrices, yet growth on a planar substrate has shown opposite behavior [23, 25, 32]. The effects of collagen/ECM stiffness on transporters such as BCRP and MRP2 has been studied in cancer cell models [14, 33, 34], but there is little reported on how matrix stiffness affects protein expression in primary intestinal cell systems. Given the growing importance of model primary-cell culture systems to understand intestinal physiology and drug interactions, there is clear need to understand the matrix or scaffold properties that yield the most in vivo-like drug transport behavior.

In this work, we characterize a conventional scaffold system and a new gradient cross-linked scaffold method for the measurement of drug transport in primary, human small intestinal epithelium [24, 25, 35–39]. Collagen was used as a substrate in both systems since it is the most abundant ECM in the intestine [21]; however, the two systems possessed a scaffold thickness many orders of magnitude different. Stem cells obtained from human small intestine were cultured on a conventional scaffold consisting of a thin collagen film over a porous membrane or on a newly developed scaffold comprised of a thick collagen layer possessing a gradient of chemical cross-linking. The presence of proliferative and differentiated cell lineages was evaluated. The ability of cells to form intercellular tight junctions was evaluated using immunohistochemical staining, and monolayer integrity was assessed by transepithelial electrical resistance (TEER) and permeability. Quantification of specific transporter protein expression was performed by quantitative targeted absolute proteomics (QTAP) by selected reaction monitoring (SRM) nanobore liquid chromatography-tandem mass spectrometry (nanoLC-MS/MS). Lastly, the movement of drugs across the monolayers by both transcellular and paracellular transport was evaluated to assess the platforms’ utility for pharmacological assays.

## Results and discussion

### Characterization of confluent small intestine monolayers on the conventional and gradient cross-linked scaffolds

**Fig. 1:**
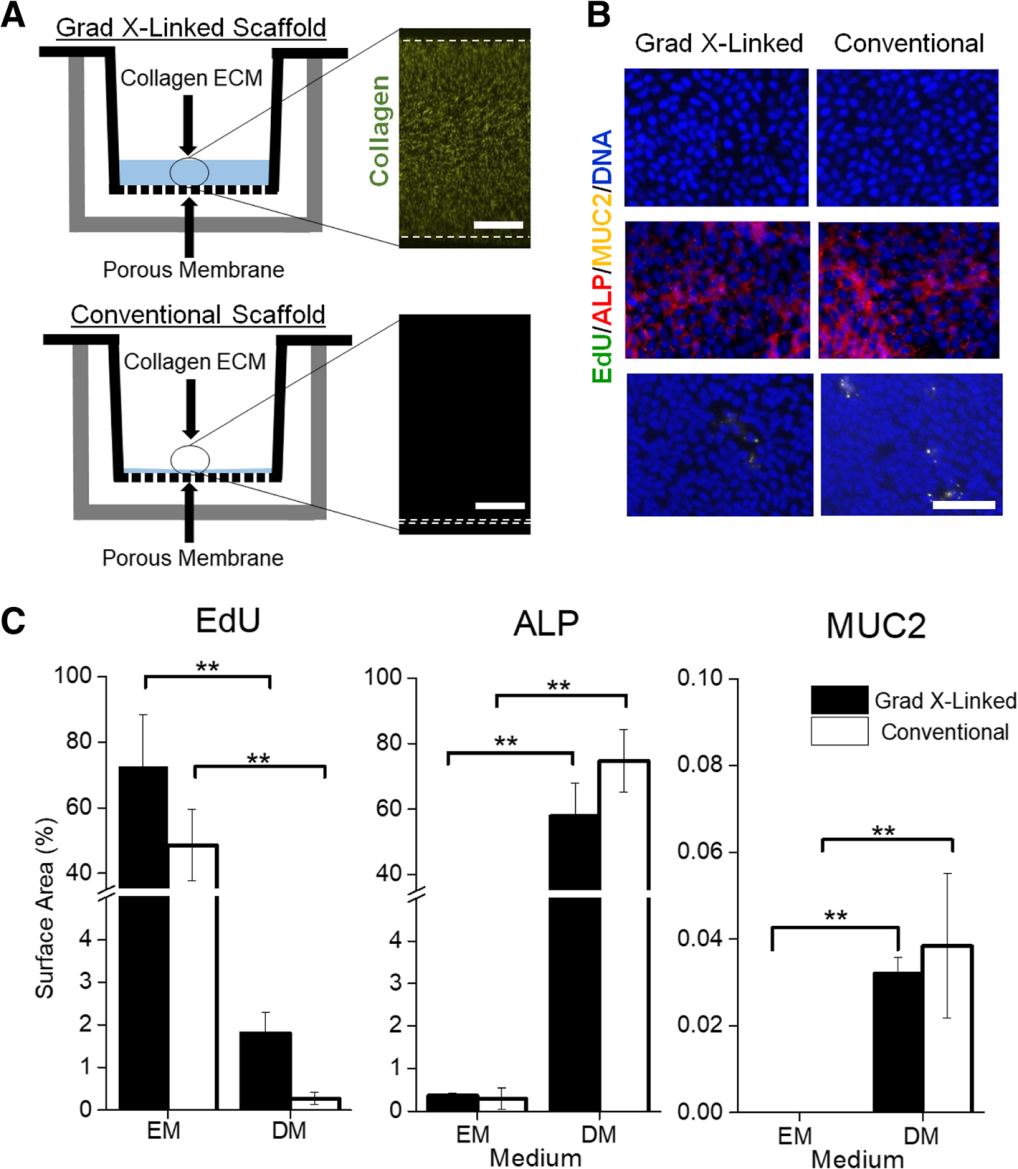
Evaluation of human, small intestinal monolayer systems. **a** Schematic and fluorescence side images of the gradient cross-linked scaffold (top) and conventional scaffold overlaying a porous membrane (bottom). Scale bar = 300 μm. **b** Fluorescence images of the monolayers at day 10 stained for EdU (green), ALP (red), Muc2 (yellow), and nuclei (blue). Scale bar = 100 μm. **c** Quantification of Edu, ALP, and Muc2 on day 5 and day 10 of culture as a percentage of the Hoechst 33342 (nuclei stain) positive area (*n* = 3). Edu, ALP, and Muc2 expression was not statistically different between the two monolayer systems in EM or DM

**Fig. 2:**
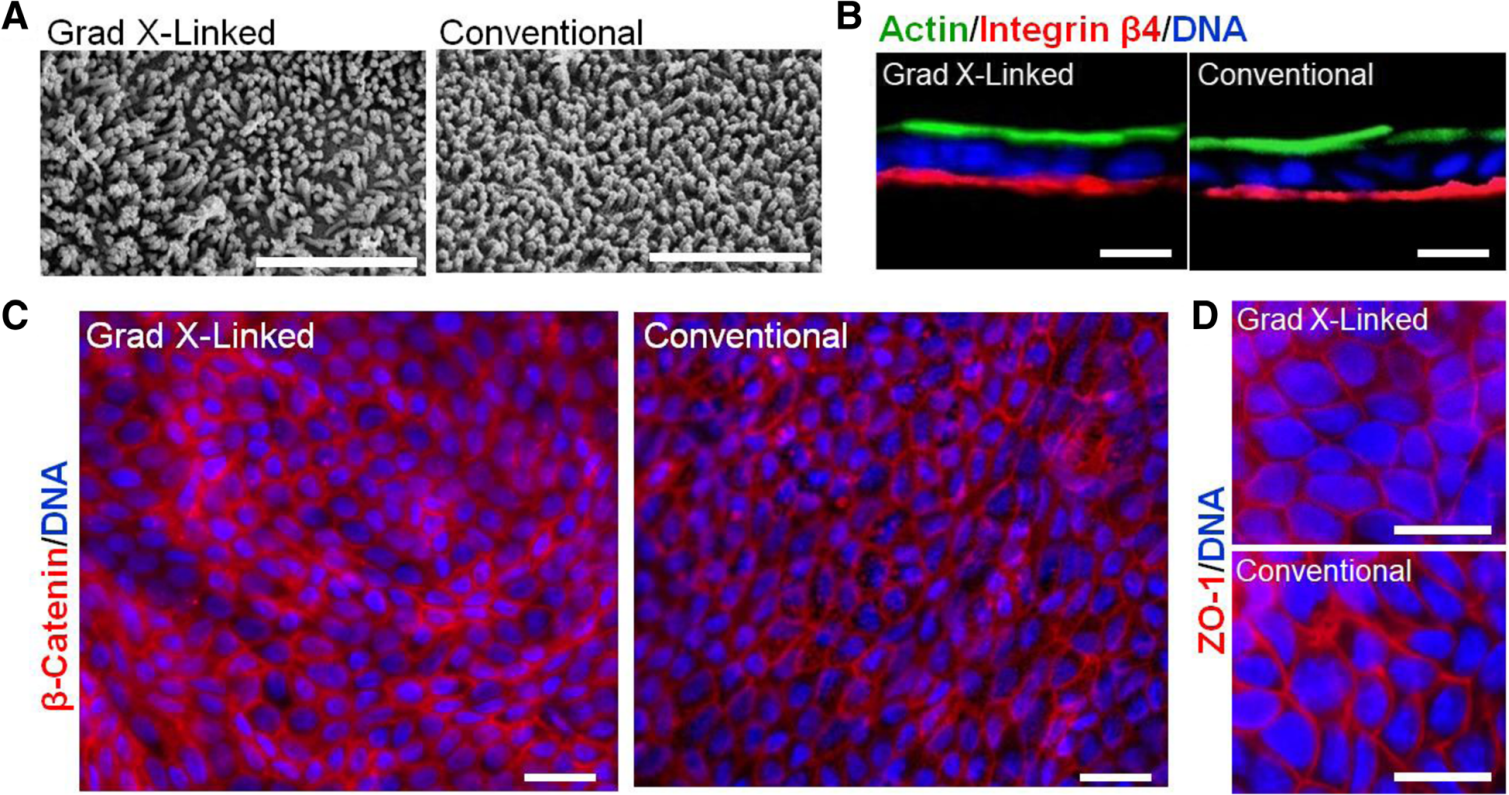
Characterization of confluent monolayer cultures. **a** Electron microscopy images of the monolayer surfaces covered with microvilli at day 10. Scale Bar = 4 μm. **b** A cross-section of the monolayers stained for F-actin (green) and integrin β4 (red). Scale bar = 10 μm. **c** Fluorescence images of the monolayers at day 10 stained with a cell adhesion protein β-Catenin (red) and Hoechst 33342 (nuclei). Scale bar = 10 μm. **d** Monolayer staining of a tight junction protein, ZO-1 (red), and Hoechst 33342 (nuclei, blue). Scale bar = 10 μm

While the two, intestinal-cell, culture systems possess a number of technical distinctions, a dominate difference is the very different surface stiffness presented to the cells (Fig. 1a, Additional file 1: Table S1). The gradient cross-linked scaffold (1.2 mm thick) possessed a stiffness of 230 ± 140 Pa, closely resembling in vivo intestinal stiffness (640 ± 340 Pa) [40]. In contrast, the conventional scaffold employing a thin (< 900 nm) film of collagen exhibited a stiffness (1.50 MPa ± 0.27 MPa) more than 2000-fold greater than that of the gradient-linked scaffold or in vivo intestine and close to that of the porous membrane alone [41, 42]. Cells were initially plated on both surfaces in expansion medium (EM), a medium rich in growth factors (Wnt-3A, R-spondin 3, and noggin) to promote stem cell proliferation and enable formation of a visually confluent monolayer (Fig. 1b, Additional file 1: Figure S1). Since transport of nutrients and drugs is accomplished predominantly by the fully differentiated enterocytes of the intestinal epithelium, differentiation medium (DM), a medium lacking growth factors, was used to replace EM in order to cause the monolayer to fully differentiate. To assess differentiation before and after media exchange, the cells were stained for Muc2 (goblet cells) and alkaline phosphatase (ALP, enterocytes). After 5 days in DM, both Muc2 and ALP staining were significantly greater for both the conventional scaffold and gradient cross-linked scaffold systems compared to cellular monolayers in EM at day 5 (Fig. 1c, Additional file 1: Figure S1). The Muc2 and ALP area coverage on the gradient cross-linked scaffold was not significantly different from that on the conventional scaffold. In vivo large numbers of microvilli cover the surface of enterocytes [43]. By SEM, both monolayer systems demonstrated a dense layer of microvilli on the luminal cell surface with a density of 3.8 ± 0.7 × 10^5^ and 2.6 ± 0.3 × 10^5^ microvilli/cm^2^ (*n* = 3) on the gradient cross-linked scaffold and conventional scaffold, respectively (Fig. 2a). These values were not significantly different (*p* = 0.08). These findings demonstrate that both monolayer systems had comparable microvillus density to that found in vivo of 4.4 ± 5.0 × 10^5^ microvilli/cm^2^ [44–46]. Actin, a protein expressed at high concentration within microvilli [47], was localized to the luminal cell surface of the monolayers on both culture platforms (Fig. 2b). In contrast, integrin-β4, which functions in the formation of ECM contacts [26, 47], was localized to the basal region of the cells on both culture platforms (Fig. 2b). These data suggest that both cell monolayer formats possessed large numbers of enterocytes with appropriate luminal-to-basal polarity supporting their suitability for transport assays.

To function effectively for transport assays, cells within the intestinal epithelial monolayer must form high-resistance contacts to prevent leakage between luminal and basal reservoirs [21, 48]. In vivo this is accomplished by formation of intercellular tight junctions containing proteins such as ZO-1 and cell-cell adhesion proteins, such as β-catenin [49]. Both β-catenin and ZO-1 were readily visualized at the interface between cells on both platforms suggesting that both culture formats might be competent to form monolayers impermeable to small molecules (Fig. 2c, d).

### Barrier function and monolayer integrity

**Fig. 3:**
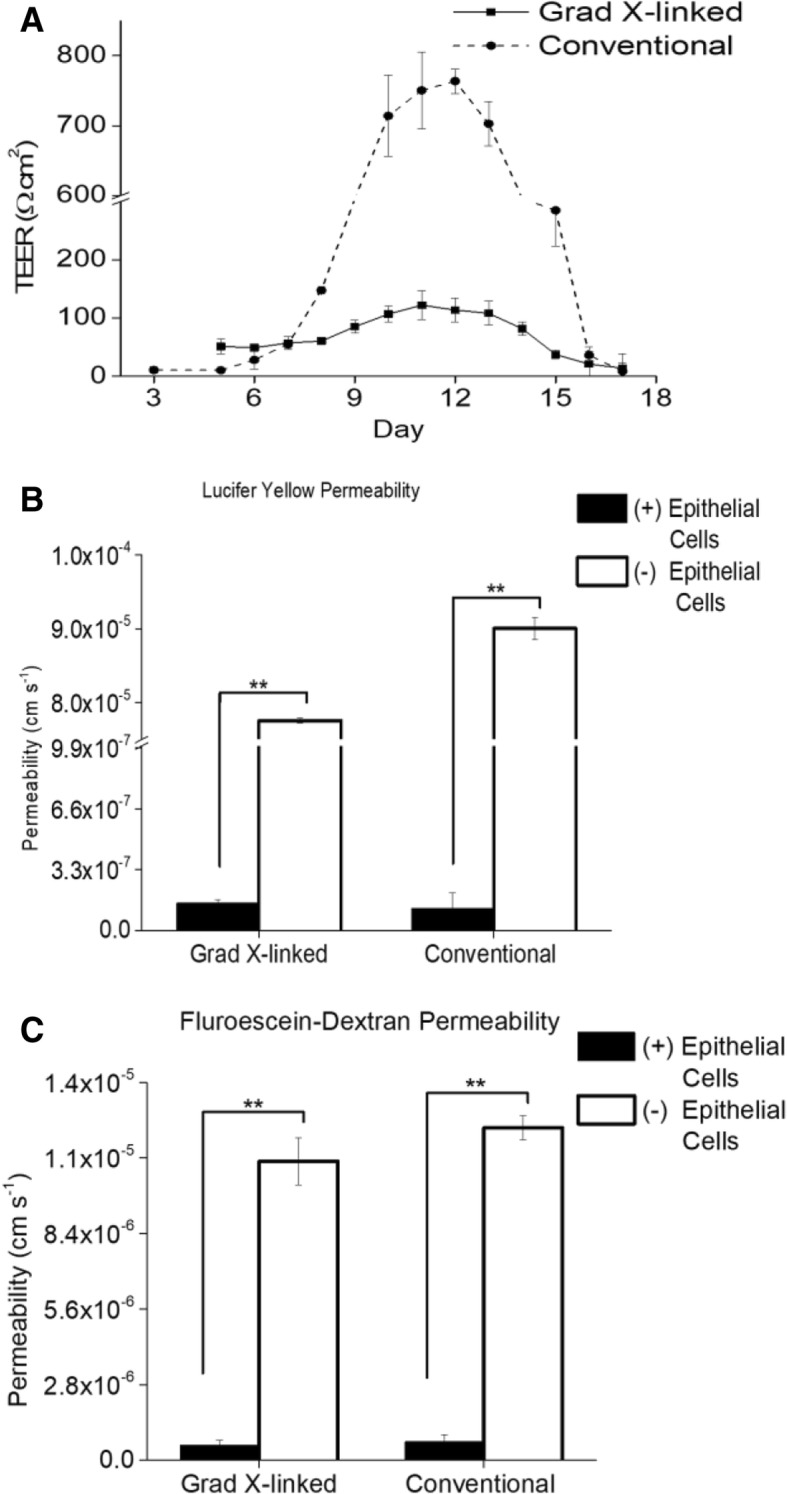
Evaluation of barrier function and monolayer integrity. **a** TEER measurements over 18 days in both monolayer culture systems (*n* = 3). **b** LY permeability values (*n* = 3) and **c**) fluorescein-dextran permeability values (*n* = 3) through the monolayers on the gradient cross-linked scaffold and conventional scaffold compared to the permeability of LY and fluorescein-dextran of the gradient cross-linked scaffold in the absence of cells (*n* = 3) and conventional scaffold in the absence of cells (*n* = 3) on day 10 of culture. The LY and fluorescein-dextran permeability values through the gradient cross-linked scaffold and conventional scaffold in the absence of cells were not statistically different

The ability of the monolayer to act as a barrier to the movement of ions, small and larger molecules was evaluated by measuring TEER over a period of 18 days. Monolayers differentiated on the gradient cross-linked scaffold possessed a maximal TEER of 120 ± 30 Ω cm^2^ (*n* = 3) at day 11 in culture. In contrast, the TEER for the monolayers on the conventional scaffold achieved a significantly greater value of 760 ± 20 Ω cm^2^ (*n* = 3) at day 12 (*p* < 0.001, Fig. 3a). The TEER value for Caco-2 cells has been reported as 250 − 4000 Ω cm^2^ while the in vivo intestine has a reported TEER value of 50–100 Ω cm^2^ [4, 50]. Thus, the TEER for the gradient cross-linked scaffold system is more representative of that found in vivo suggesting that the low-stiffness, gradient cross-linked scaffold may provide an in vitro ECM platform more similar to that found in vivo than that produced on stiff surfaces.

While TEER is an important metric for membrane integrity, its value can vary considerably across different regions of the intestine based on that region’s ion transport functions, and is most reflective of ion permeability rather than that of large molecules such as drugs [48, 51]. For this reason the ability to block diffusion of non-transported molecules such as Lucifer yellow (LY) or fluorescent dextrans is often used to assess the barrier function of the intestinal epithelium [25, 51]. Both LY and fluorescein-dextran (70 kDa) were added to the luminal reservoir (top) and their movement into the basal reservoir (bottom) measured after 3 h. The permeability of LY and fluorescein dextran through confluent, differentiated monolayers of cells cultured on the gradient cross-linked scaffold and conventional scaffold was low and not significantly different (*n* = 3) (Fig. 3b,c). Previous reports of LY and fluorescein-dextran (70 kDA) permeability for Caco-2 cells are < 1 × 10^− 6^ cm s^− 1^ and (3–7) × 10^− 7^ cm s^− 1^, respectively [52, 53]. Thus, the two different monolayer systems possessed similar barrier function as that posed by Caco-2 cells. In general for a monolayer to be suitable for transport studies, the monolayer permeability to these non-transported molecules should be < 1 × 10^− 6^ cm s^− 1^ [54]. These data suggest that both monolayer culture systems exhibit high barrier integrity.

### Measurement of intestinal proteins involved in transport

Absolute quantitative proteomics via SRM nanoLC/MS-MS was used to assess the presence of proteins known to participate in the movement of drugs, metabolites, ions and amino acids across the intestinal surface as these are expected to be present in a functional intestinal epithelium [7, 55]. Protein expression levels of monolayers cultured on the conventional scaffold or gradient cross-linked scaffold were measured on day 10 and compared to that of freshly isolated crypts/villi (Table 1, Additional file 1: Figure S2). Na^+^/K^+^-*ATPase* and GGTP in both monolayer culture systems were expressed at levels similar to that in fresh tissue (Table 1, Additional file 1: Figure S2). P-gp and OCT3 expression on the two systems were also similar to that found in fresh intestinal epithelium (Table 1, Additional file 1: Figure S2). In contrast, BCRP and MRP2 were expressed at comparable levels in the monolayers on the gradient cross-linked scaffold relative to that found in fresh crypts/villi, but at much greater levels than that of the monolayers on the conventional scaffold (Table 1, Additional file 1: Figure S2). The equivalent substrate stiffness compared to in vivo intestine of the gradient cross-linked scaffold and the similar protein expression of BCRP and MRP2 in relation to the fresh crypts/villi tissue suggest that this scaffold may be a more suitable model system for assay of some transporters.

**Table 1:** Protein expression of selected transporters in both monolayer culture systems measured at day 10 of culture and in fresh crypts/villi. Concentrations (C) are reported in pmol/mg membrane fraction protein; C > 5 (++++); 5 > C > 3 (+++); 3 > C > 0.5 (++); 0.5 > C (+)

Protein	Grad X-linked Scaffold	Conventional Scaffold	Fresh Crypts/ Villi
P-gp	++++	++++	++++
BCRP	++++	+	++++
MRP1	+	+	+
MRP2	+++	++	+++
MRP3	++	++	++
OCT3	++	++	++
Na K ATPase	++++	++++	++++
GGTP	++++	++++	++++

### Functional assays

**Fig. 4:**
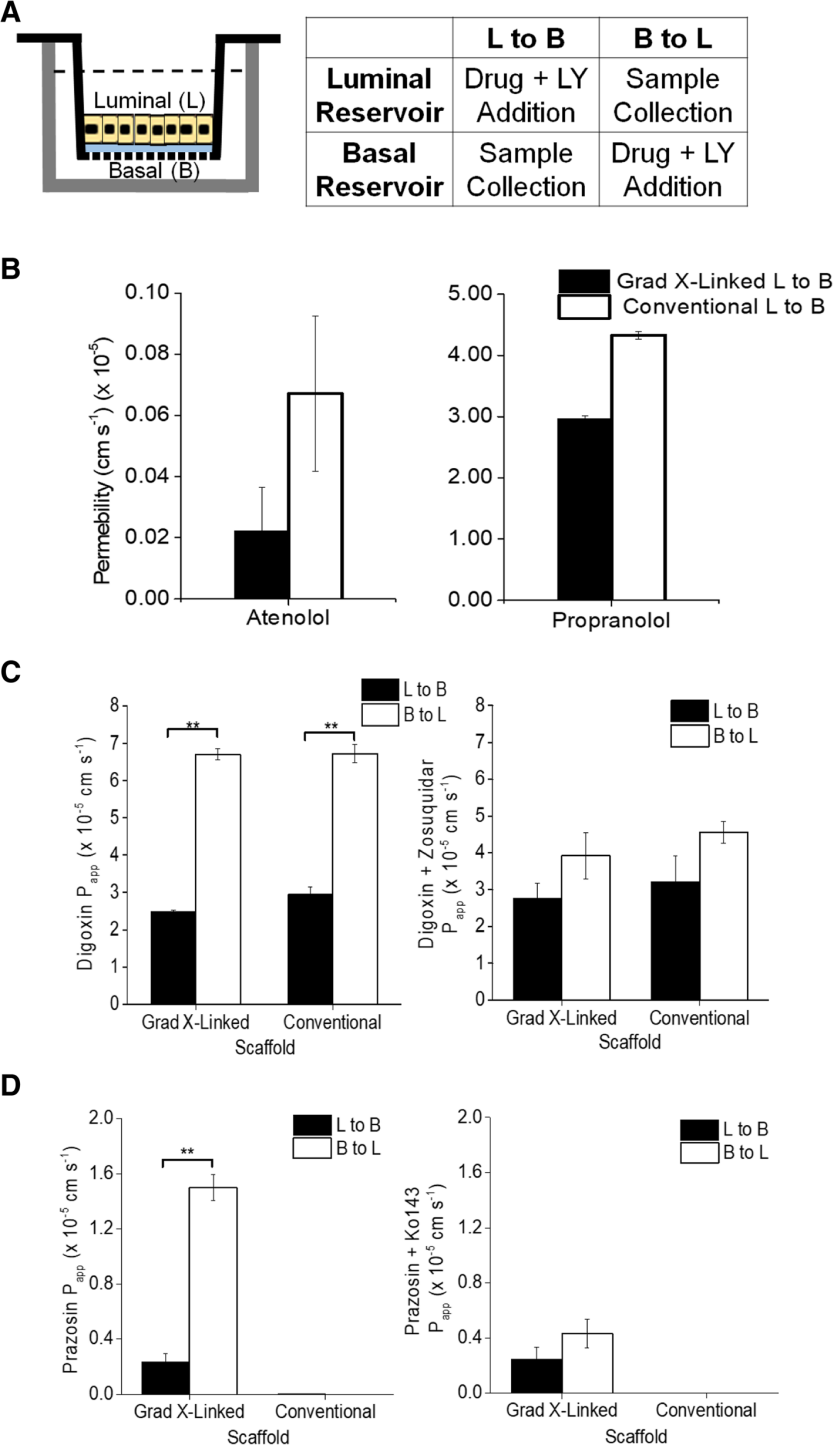
Evaluating compound transport across the monolayer platforms. **a** Experimental design of luminal to basolateral and basolateral to luminal transport assays. **b** Permeability values of the monolayer on the culture systems to atenolol (paracellular transported drug, low permeability molecule, *n* = 3) and propranolol (passive transcellular transported drug, high permeability molecule, *n* = 3). **c** Permeability of digoxin in the basal to luminal or luminal to basal direction in the two culture formats without (left panel) and with (right panel) the P-gp inhibitor zosuquidar (*n* = 3). The inhibition data was not significantly different for the four data points. **d** Permeability of prazosin in the basal to luminal or luminal to basal direction in the two culture formats without (left panel) and with (right panel) the BCRP inhibitor Ko143 (*n* = 3). The inhibition data was not significantly different for the for the two transport directions on each scaffold

To evaluate protein function, clinically relevant substrates known to be carried by three different mechanisms were assessed: atenolol (paracellular transport), propranolol (transcellular transport), and digoxin and prazosin (efflux transcellular transport). Atenolol and propranolol are antihypertensive drugs that inhibit the β-adrenergic receptor and are frequently used as model drugs for assessment of hydrophilic (atenolol) and lipophilic (propranolol) drug uptake [56]. Atenolol uptake is thought to occur passively across the intestinal epithelium between the cells, i.e. through an intercellular pathway [57]. In contrast, propranolol is thought to move by a transcellular pathway i.e. passing through cell membranes and crossing the cytoplasm [16]. In vitro studies have shown that propranolol can be actively exported as a substrate for P-gp, but the full mechanism is still unknown [58]. Atenolol and propranolol were added to the luminal compartment and samples were collected from the basal reservoir after 3 h (Fig. 4a). LY served as a control for monolayer barrier integrity. LY permeability was always less than 1 × 10^− 6^ cm s^− 1^ in these experiments. The permeability of atenolol through the monolayer on the conventional scaffold was not significantly different from that on the gradient cross-linked scaffold (Fig. 4b, *n* = 3). The atenolol permeability for both monolayers was similar to that previously reported for Caco-2 cells of [2–10] × 10^− 7^ cm s^− 1^, but substantially different from that estimated from in vivo studies (100–180 × 10^− 7^ cm s^− 1^) [59–61]. The permeabilities of propranolol through the monolayer on the gradient cross-linked scaffold and conventional scaffold were not statistically different (Fig. 4b), were comparable to that of Caco-2 cells [4–25] × 10^− 6^ cm s^− 1^, and equivalent to that measured in vivo (5–7 × 10^− 5^ cm s^− 1^) [59–63]. For the transport of these two drugs, the gradient cross-linked scaffold and conventional scaffold performed in a similar manner, although atenolol uptake was less than that reported for atenolol in vivo [60, 61].

To evaluate drug efflux and directional transport, the permeability of digoxin, a P-gp substrate, and prazosin, a BCRP substrate, was added to either the basal or luminal compartments of the two cell culture formats followed by assay of the drug in the opposite reservoir (Fig. 4a). Digoxin, a Na+/K + -ATPase inhibitor, is used in the treatment of heart disease, and prazosin, an α-1-adrenergic receptor antagonist, is used to treat hypertension [64, 65]. The permeability of digoxin from the basal to the luminal monolayer side of the gradient cross-linked scaffold (*n* = 3) and conventional scaffold (*n* = 3) was not statistically different (Fig. 4c) and was similar to that reported previously in primary human small intestinal epithelial cells [5–10] × 10^− 6^ cm s^− 1^ [16]. Digoxin had greater permeability in the basal to luminal direction in both the gradient cross-linked and the conventional scaffold with efflux ratio of 2.7 and 2.3 (*n* = 3), respectively. In the presence of P-gp inhibitor zosuquidar, the efflux ratio of digoxin reduced to 1.4 (*n* = 3) in both monolayer systems. The permeability of prazosin from the basal to the luminal monolayer side of the gradient cross-linked scaffold (*n* = 3) when cultured on the gradient cross-linked scaffold was comparable to that of previous reports for Caco-2 cell monolayers (3–5 × 10^− 6^ cm s^− 1^) [66]. Prazosin moved preferentially from the basal to luminal monolayer side with an efflux ratio of 6.5 on the gradient cross-linked scaffold and was readily blocked by a BCRP inhibitor (Ko143) decreasing the efflux ratio to 1.7. The permeability of prazosin through the monolayer on the conventional scaffold from basal to luminal compartments was below the limits of detection of the assay system (1.2 ± 0.9 × 10^− 9^ cm s^− 1^) (*n* = 3), while the permeability of prazosin through the monolayer on the conventional scaffold from luminal to basal compartments was 2.35 × 10^− 8^ (Fig. 4d), demonstrating that efflux transport did not occur. This finding was likely due to the very low level of BRCP protein expressed in the monolayers on the conventional scaffold relative to that of fresh tissue or the monolayers on the gradient cross-linked scaffold. These data suggest that developing in vitro systems that incorporate features most reflective of the in vivo ECM is important in order to accurately mimic in vivo intestinal epithelial transport.

## Conclusions

Confluent monolayers of human small intestinal epithelial cells were formed using two culture formats: a thick gradient cross-linked collagen scaffold or a more conventional format with a thin film of collagen. While the two systems incorporated a number of differences, the most prominent was the > 1000-fold difference in scaffold stiffness (Additional file 1: Table S1). The height of the medium above the monolayer in the two culture systems was comparable (9 mm gradient cross-linked vs 10 mm conventional) and COMSOL modeling suggested similar O_2_ saturation in the two systems (Additional file 1: Figure S3). COMSOL modeling also suggested that access of nutrients to the cells from the luminal and basal reservoirs was indistinguishable for the two culture formats so that the different pore densities in porous membranes was unlikely to impact cell behavior. Finally, the collagen layer/coatings most likely masked any surface chemical differences between the porous membranes of the two culture formats. Despite the vast difference in surface stiffness, the cells on the two culture formats were remarkably similar except in their transport properties. Both formats displayed alkaline phosphatase activity and possessed mucin 2, indicating the presence of enterocytes and goblet cells, respectively. Neither LY nor fluorescein-dextran permeated the cell monolayers suggesting that both monolayer systems yield tissue with high barrier integrity. Additionally, TEER values of both monolayers were equivalent to or higher than that of in vivo intestine suggesting that ions did not readily cross the confluent monolayers. Measurement of protein expression in cells cultured on the gradient cross-linked scaffold and conventional scaffold demonstrated the presence of multiple transporter proteins at levels similar to those present in freshly isolated crypts/villi. A notable exception to these similarities was the diminished expression of the BCRP and MRP2 proteins in the cells on the conventional scaffold compared to that in cells cultured on the gradient cross-linked scaffold and crypts/villi of the small intestine. The monolayer on the conventional scaffold failed to transport the BCRP substrate prazosin from the basal to the luminal compartments although prazosin was transported by the monolayers on the gradient cross-linked scaffold. Given that MRP2 protein expression was low in the cells on the conventional scaffold, it is likely that MRP2 substrates would not be transported by these cells but would be by cells on the gradient cross-linked scaffold since those monolayers possessed similar MRP2 expression to that of the in vivo intestine. Based on the comparable levels of P-gp expression, it was perhaps not surprising that the P-gp substrate digoxin was directionally transported by cells on both culture systems. While we can not rule out alternative mechanisms, the most likely source of the drug transport differences between the two cell monolayers is the scaffolding stiffness. It has been shown that ECM stiffness sensed by the cell are propagated, amplified, and transduced into signaling cascades to lead to transient or sustained cellular responses, ultimately affecting protein expression and amplification and potentially transporter behavior [67]. This is likely the case for a subset of transporter proteins in the intestinal epithelium. This work highlights the importance of developing in vitro scaffold systems for the intestine to recapitulate *lamina propria* properties as close as possible to yield intestinal models displaying the greatest repertoire of physiologic properties.

## Methods

### Materials

Rat tail type I collagen, Transwell™ inserts, 12-well polystyrene tissue culture dishes, and 12-well polycarbonate filters were purchased from Corning (Corning, NY). Human collagen (type I) was obtained from Advanced Biomatrix (Carlsbad, CA). Collagenase (type IV) was from Worthington Biochemicals (Lakewood, NJ). Gastrin was obtained from Anaspec (Freemont, CA). *N*-acetyl cysteine was from MP Biomedicals (Santa Ana, CA). Murine EGF was obtained from Peprotech (Rock Hill, NJ). Primocin was purchased from InvivoGen (San Diego, CA). SB202190 was obtained from Selleckchem (Houston, TX). Nicotinamide, A83–01, fluorescein-dextran (70 kDa), atenolol, metoprolol, propranolol, prazosin, doxazosin, digoxin, zosuquidar, and digitoxin were acquired from Sigma-Aldrich (St. Louis, MO). Y-27632 and N-[N-(3,5-Difluorophenacetyl)-L-alanyl]-S-phenylglycine t-butyl ester (DAPT) was from ApexBio Technology (Houston, TX). Red alkaline phosphatase substrate kit was obtained from Vector Labs (Burlingame, CA). Rabbit α-Muc2, ZO-1, and integrin β4 were obtained from Santa Cruz Biotechnology (Dallas, TX). Donkey serum and donkey anti-rabbit or mouse IgG conjugated with Alexa Fluor 488 were from Jackson Immunoresearch (West Grove, PA). Ko143 was purchased from Cayman Chemical (Ann Arbor, MI). All other reagents were from Thermo Fisher Scientific (Waltham, MA).

### Cell culture media composition

Expansion media (EM) for cell culture contained advanced DMEM/F12 medium, L-WRN conditioned medium, HEPES (10 mM, pH 7.5), EGF (50 ng/mL), *N*-acetyl cysteine (1.25 mM), B27 (1 ×), GlutaMAX (2 mM), prostaglandin E2 (10 nM), nicotinamide (10 mM), gastrin (10 nM), SB202190 (3 μM), penicillin (100 unit/mL), streptomycin (100 μg/mL), primocin (100 μg/mL), and Y-27632 ROCK inhibitor (10 μM). EM lacking Y-27632 ROCK inhibitor was used after the initial 48 h of culture. L-WRN conditioned medium was prepared by culture of L-WRN cells (ATCC #CRL-3276) as previously described [68]. This cell line secretes Wnt-3A, R-spondin 3, and noggin into the medium which was harvested and used a source of growth factors for culture of the small intestinal epithelial cells. Wnt-3A, R-spondin 3, and noggin were used at concentrations of 45, 25, and 25 ng/mL, respectively, to culture the epithelial cells [23]. Differentiation media (DM) contained DMEM/F12 medium, HEPES (10 mM, pH 7.5), GlutaMAX (2 mM), primocin (100 μg/mL), *N*-acetyl cysteine (1.25 mM), EGF (50 ng/mL), DAPT (20 ng/mL), and A83–01 (500 ng/mL).

### Preparation of a gradient cross-linked scaffold on a porous membrane for cell culture

A gradient cross-linked scaffold on a porous membrane was prepared according to a published protocol [25]. Briefly, neutralized collagen mixture (200 μL, 1 mg/mL rat tail, Type 1) was added to the upper reservoir of a Transwell insert (BD Falcon #353180, PET membrane, 0.9 cm^2^ cell culture area, 1.6 × 10^6^ pores/cm^2^), and then incubated at 37 °C for 1 h. Following incubation, phosphate buffered saline (PBS, 1 mL) was added to the upper and lower reservoir of each Transwell. The Transwell inserts were placed at 4 °C for 30 min. The PBS in the lower reservoir was replaced with 1-ethyl-3-(3-dimethylaminopropyl) carbodiimide hydrochloride (EDC, 353 mM) and *N*-hydroxysuccinimide (NHS, 88 mM) in 2-morpholinoethanesulfonic acid (MES, pH 5.5) to form a gradient of cross-linked collagen along the depth of the scaffold as described previously [25]. The crosslinking ensures the long-term mechanical integrity of scaffolds without cell-induced contraction. The inserts and plates were maintained at 4 °C for 40 min. The Transwells were removed from the culture plate and immediately placed in deionized water for 24 h. The inserts were then sterilized with 75% ethanol, rinsed with PBS × 3, and stored in PBS at 4 °C until use.

### Preparation of a conventional scaffold on a porous membrane for cell culture

A conventional scaffold on a porous membrane was prepared by following the manufacturer’s protocol (Corning, Corning, NY). Briefly, rat tail collagen (100 μL, 0.1 mg/mL) was diluted in ethanol (70%) and the mixture was added to the upper reservoir of a Transwell cassette (Corning Life Sciences #3401, 1.12 cm^2^ cell culture area, 1.6 × 10^8^ pores/cm^2^) to form a thin collagen film on the membrane. The Transwell inserts and plates were dried inside a laminar flow hood for 3 h and sterilized under UV light for 1 h. After sterilization, the upper reservoir and lower reservoir of the Transwells were immediately coated with human collagen (1 mL, 0.01 mg/mL solution) in sterile PBS. The plates were placed in 37 °C for 24 h. All liquid was then aspirated from the cassette, the upper and lower reservoirs of the Transwell were washed with PBS × 3, and the cells were plated immediately afterwards.

### Primary human small intestine monolayer cell culture

Human small intestinal (jejunum) were obtained from a gastric by-pass procedure at UNC Hospitals with patient consent under an approved protocol (UNC IRB #14–2013). This tissue was acquired from a single donor and used for all experiments in this work. Multiple technical replicates (*n* > 3) were used in all experiments. Crypts/villi were isolated from the tissue following a previously published protocol [23, 24]. Briefly, the tissue was cut longitudinally to expose the luminal and basal sides of the tissue incubated with ethylenediaminetetraacetic acid (EDTA, 2 mM) and dithiothreitol (DTT, 0.5 mM) in buffer (5.6 mM Na_2_HPO_4_, 8.0 mM KH_2_PO_4_, 96.2 mM NaCl, 1.6 mM KCl, 43.4 mM sucrose, 54.9 mM D-sorbitol, pH 7.4) for 90 min at 25 ^°^ C. The crypts/villi were dissociated by vigorous shaking and then collected by centrifugation. The supernatant was removed and replaced with EM. The crypts/villi in EM were plated on a thick non-cross-linked collagen hydrogel (1 mg/mL, rat tail, pH 7.4) on a polystyrene tissue culture plate as described previously [23, 24]. After 5 days, the cells were passaged as previously described [24]. Briefly, the cells along with the collagen hydrogel were removed from the plate surface using a 1000 μL pipette tip and transferred into a 15 mL conical tube containing EM (1 mL) and 500 U/mL of Type IV collagenase. The hydrogel with attached cells was fragmented by repeated pipetting using a 1000 μL pipette tip and then incubated at 25 °C for 10 min. This suspension was centrifuged at 600×g for 1 min and the pellet was washed with PBS (5 mL). The mixture was then re-centrifuged at 600×g for 1 min. The pellet was incubated with EDTA (0.5 mM) and Y-27632 (10 μM) in PBS at 37 °C for 10 min. The monolayers were defragmented by repetitive pipetting up/down by a 200 μL pipette tip 60 times. The cells were re-suspended in EM and expanded until P5. After P5, the cells were plated on either the gradient cross-linked scaffold or conventional scaffold at a cell density of 1.6 × 10^5^ cells cm^− 2^ as described above or stored in liquid nitrogen. If the cells were plated on the gradient cross-linked scaffold or conventional scaffold, the top (luminal) and bottom (basal) compartments of the Transwell cassette contained 1 and 2 mL of EM, respectively. The height of media fluid in the gradient cross-linked scaffold is 9 mm, and the height of media fluid in the conventional scaffold is 10 mm. After 48 h of culture, EM without Y-27632 was placed in both cassette compartments (luminal and basal). The EM was replenished every 48 h for 5 days followed by addition of DM (in place of EM) on day 5. The DM was changed every 24 h until the monolayers were utilized for experiments. Cells were maintained at 37 °C in 5% CO_2_ incubator. If cells were stored in liquid nitrogen, 1 M cells were snap frozen at P5 in a solution (1 mL) containing 50% EM, 40% FBS, and 10% DMSO in cryogenic storage vials (Thermo Fisher Scientific, Waltham, MA). Cells were karyotyped (KaryoLogic, Inc., Morrisville, NC) at passage number P15. Twenty cells from each sample (*n* = 3) were analyzed to confirm that the cells possessed a normal human karyotype (Additional file 1: Figure S4). Cells were never used beyond P15.

### Stiffness measurements

The stiffness of the gradient cross-linked and conventional scaffolds were measured in PBS using atomic force microscopy (AFM, Asylum Research MFP3D) as previously described [25]. Prior to AFM analysis, the scaffolds were cut and removed while remaining attached to the permeable membrane, placed on a microscope glass slide and secured with an O-ring made from polydimethylsiloxane (Dow Corning, Midland, MI). A 2.25 μm polystyrene spherical bead mounted on a 30 pN/nm silicon nitride cantilever (Novascan Technologies, Inc., Ames, IA) was used to apply a force (100–1000 pN) perpendicularly to the scaffold surfaces. The cantilever’s displacement in response to the applied force was recorded. For both scaffold systems, force vs. displacement curves were determined ≥5 different positions across the surface with 5 measurements taken at each position. A thermal tune method was used before measuring the stiffness of each scaffold surface to determine the spring constant of each cantilever (total average spring constant of 2 cantilevers: 31.99 ± 4.73 pN/nm). Stiffness was determined by using the Hertz model to fit force vs. indentation curves [69].

### Evaluation of small intestinal epithelial monolayer integrity

Monolayer integrity was evaluated by TEER and the permeability to fluorescein-dextran M.W. 70 kDA and Lucifer yellow (LY) M.W. 442.2 g/mol. Neither LY nor fluorescein-dextran are transported by the intestinal epithelium [25, 54]. The TEER was measured using an EVOM^2^ epithelial Volt/Ohm meter (World Precision Instruments, Sarasota, FL). The raw resistance measurements of the monolayers were subtracted from that of the average resistance (*n* = 3) of the collagen layer without cells and normalized by multiplying the cell culture area of the well to provide a TEER in units of Ω cm^2^ [48]. The average TEER (*n* = 3) with a single standard deviation (SD) is shown (average ± SD). The permeability of LY (500 μM in DM, *n* = 3) and fluorescein-dextran (1 mg/mL in DM, *n* = 3) was determined by adding the compound to the luminal side of the monolayer and collecting samples from the basal side after 3 h. The amount of LY (ex: 428 nm, em: 520 nm) and fluorescein-dextran (ex: 490 nm, em: 520 nm) in the basal side was determined by measuring their fluorescence in a microplate reader (Spectramax M5, Molecular Devices Corporation, San Jose, CA).

The luminal-to-basal apparent permeability (P_app_) were calculated according to the following equation: P_app_ = (V/(A x C_i_)) x (C_f_/T) where V is the volume of the basal chamber (mL), A is the area of epithelial layer (cm^2^), C_i_ is the initial concentration of the molecule (μM), C_f_ is the final concentration of the drug (μM), and T is the assay time (s) [70]. The average P_app_ with a single standard deviation is shown (average ± SD).

### EdU, ALP, and MUC2 fluorescence stains and quantification

The small intestine monolayers (*n* = 3) were stained at days 5 and 10 to evaluate cell proliferation and differentiation over time. A 5-ethynyl2-deoxyuridine (EdU) stain was used to mark cells in S phase of the cell cycle. The cells were incubated with EdU (10 μmol/L) in EM or DM (day 5, 10 respectively) at 37 °C for 6 h. Then, the cells were stained with a Click-iT EdU Alexa Fluor 647 imaging kit according to the manufacturer’s directions (Thermo Fisher, Waltham, MA). Enterocyte presence was evaluated by assessing alkaline phosphatase (ALP) activity. The cells were washed with PBS × 3, incubated for 30 min at 37 °C with ALP substrate (Vector red alkaline phosphatase substrate kit; Vector Laboratories, Burlington, CA) in tris buffer (0.15 mol/L, pH 8.4) for 30 min, and then the monolayers were fixed in paraformaldehyde (4%) for 15 min. The fixed cells were analyzed for the presence of mucin-2 (Muc2) by immunostaining with a rabbit anti-mucin2 primary antibody (α-muc2, 1:200, Santa Cruz Biotechnology, Dallas, TX, #sc-15,334) followed by an Alexa Fluor 488 α-rabbit secondary antibody (1:500, Jackson Immunoresearch, West Grove, PA, # 711–545-152). DNA was stained by incubating the cells with Hoechst 33342 (2 μg/mL, Sigma Aldrich, St. Louis, MO, #B2261) for 15 min at 25 °C.

The monolayers were imaged using a Nikon Eclipse TE300 inverted epifluorescence microscope. Images were acquired with a 10× objective (N.A. = 0.3). S-phase cells stained with the Click-iT EdU stain were imaged using a CY5 filter (excitation filter 604–644 nm, emission 672–712 nm). The ALP stain was imaged with a Texas Red filter (excitation filter 542–582 nm, emission 604–644 nm). Muc2 immunofluorescence was visualized with a fluorescein filter (excitation filter 450–490 nm, emission 520 nm long pass). DNA was stained with Hoechst 33342 were analyzed using a DAPI filter (excitation filter 352–402 nm, emission 417–477 nm). Images were empirically thresholded using Image J (https://imagej.nih.gov/ij/). The percent surface area of each image displaying supra-threshold fluorescence from the EdU, ALP or Muc2 stains was measured. These areas were then normalized by dividing by the area of the Hoechst fluorescence (DNA stain) resulting in a number that expresses the percentage of the monolayer area positive for the EdU-incorporation, ALP activity, or Muc2 protein. Five different locations in the same monolayer were used to generate the image data and this experiment was repeated (*n* = 3). The average with a single standard deviation is shown (average ± SD).

### Other fluorescent staining

The monolayers were also stained for expression of β-catenin, filamentous actin (F-actin), integrin β 4, and ZO-1. For all antibodies, except ZO-1, the cells were first fixed with paraformaldehyde (4%) for 15 min and treated with 0.5% triton X-100 for 20 min at 37 °C to facilitate diffusion of the labeling reagents into the cells. For ZO-1, the cells were fixed with methanol (4 ^°^ C) and incubated at − 20 °C for 20 min. The cells were blocked with 10% donkey serum (Jackson Immunoresearch, West Grove, PA, # 017–000-121) for 1 h at 20 °C. The cells were then incubated in primary antibody at 4 °C for 24 h. After 24 h, the samples were stained with a secondary antibody for 45 min at 20 °C. Primary antibodies used were rabbit anti-integrin β4 (1:200, Santa Cruz sc-9090; Santa Cruz Technology, Santa Cruz, CA), rabbit anti-ZO-1 (1:100, Proteintech 21,773–1-AP; Wuhan, China), and rabbit anti-β-catenin (1:200, Santa Cruz sc-7199, Santa Cruz Technology, Santa Cruz, CA). The secondary antibodies were donkey anti-rabbit immunoglobulin G conjugated with either Alexa Fluor 488 or 594 (1:500, Jackson Immunoresearch 711–545-152 or 711–585-152, West Grove, PA) or Alexa Fluor 488 phalloidin stained F-actin (Thermo Fisher A12379). When staining cross-sections of the monolayer for integrin β4 and actin, the samples were fixed in paraformaldehyde (4%) for 15 min, rinsed with PBS, and incubated overnight in 30% sucrose at 4 °C. The monolayer was then submerged in a cryo-embedding medium (OCT). The frozen tissue block was sectioned into 10-μm thick films with a cryostat.

To measure the thickness of collagen in the gradient cross-linked scaffold and conventional scaffold, collagen was plated in each culture system as previously described in this manuscript. Collagen was incubated with NHS-Fluorescein in PBS (0.25 μg/mL, Thermo Fisher, Waltham, MA, #46410) at 25 °C for 24 h, and immediately placed in deionized water for 24 h. Images were immediately acquired by a confocal laser scanning microscope (Fluoview FV3000; Olympus, Waltham, MA) and visualized with a fluorescein filter (excitation filter 467–498 nm, emission 505–548). Images shown in Fig. 1a were acquired with a 20× objective (N.A. = 0.45). The collagen layer in the conventional scaffold was not visible using a 30x objective (N.A. = 1.05) which has a Z-resolution of 900 nm [71].

### Electron microscopy

Scanning electron microscopy (SEM) was utilized to evaluate the presence of microvilli. The monolayers were fixed with paraformaldehyde (4%) for 15 min. Then the monolayers were incubated in deionized water for 24 h. After 24 h, the monolayers were placed into 25, 50, and 75% ethanol sequentially for 1 h. The monolayers were placed in a critical point dryer (PVT-3, Tousimis Semidri, Rockville, MD) to dehydrate the samples. The monolayers were then coated with metal (10 nm gold; Cresington Scientific Instruments) and imaged by a scanning electron microscope (FEI Quanta 200 ESEM, FEI Company, Hillsboro, OR).

### Proteomic analysis

QTAP nanoLC-MS/MS methods previously published were used to quantify protein expression of drug transporters, NaK ATPase, and GGTP in the monolayers and fresh crypts/villi [72, 73]. The monolayer samples were prepared from 10 million cells that were pooled and homogenized in hypotonic buffer containing 10 mM tris HCl (pH 7.4), 10 mM NaCl, 1.5 mM MgCl_2_. The samples were centrifuged (10,000 rcf, 4 °C) and the protein in the supernatant was pelleted by ultracentrifugation (100,000 rcf, 4 °C). The resulting membrane fraction was re-suspended in fresh PBS for analysis. Prior to nanoLC-MS/MS analysis, a stable isotope-labeled proteotypic tryptic peptide standard of known concentration was added to the membrane fraction samples. Absolute quantification was determined from the ratio of two SRM peak area signals of the analyte summed to two corresponding signals of the stable isotope-labeled peptide standard summed, equality of response between the analyte (unlabeled) and labeled peptides being assumed.

### Functional transport assays

All transport assays were performed by adding LY (500 μm) and the drug to the luminal or basal reservoirs of the monolayer (*n* = 3). Samples were collected after 3 h. LY was added to ensure that monolayer integrity remained intact throughout the assay. Additionally, TEER was measured before and after the assay to further verify that monolayer integrity remained constant throughout the assay. Monolayers with a TEER > 90 Ω cm^2^ or 300 Ω cm^2^ were used for the gradient cross-linked scaffold and conventional scaffold, respectively. These values were established based on the TEER at which the monolayer was impermeable to LY. For atenolol and propranolol, the compounds were added to the luminal compartment, and fluid was collected from the basal compartment. Bidirectional transport of digoxin and prazosin in the presence and absence of a known selective inhibitor was evaluated. Zosuquidar and Ko143 were added to inhibit digoxin transport and prazosin transport, respectively, 2.5 h after addition of the substrate. Samples were collected 30 min after inhibitor addition for a totally assay time of 3 h.

The concentration of the drug in the collected sample was determined using HPLC-triple quadrupole mass spectrometry (LC-MS/MS, PE Sciex API 3000, AB Sciex) with multiple reaction monitoring (MRM). A C-18 analytical column with a linear gradient from 100% water with 1% formic acid to 80% acetonitrile with 1% formic acid over 8 min (0.3 mL/min) was used for the separation. Metoprolol was used as an internal standard for atenolol and propranolol samples. Digitoxin was used as an internal standard for digoxin samples. Doxasozin was used an internal standard for prazosin samples. All internal standards were added to samples just prior to LC-MS/MS analysis. Eluted species were subjected to electrospray ionization (positive mode) and transitions of 267.2/190 (m/z of precursor ion/product ion) for atenolol, 260.2/116.1 for propranolol, 268.2/116.1 for metoprolol, 798.5/651.5 for digoxin, 782.5/635.5 for digitoxin, 384.3/247.2 for prazosin, and 452.3/344.3 for doxazosin were identified [56, 74, 75]. Sample concentrations were quantified using the standard curve and normalized to the signal of the appropriate internal standard. The drug apparent permeability was calculated according to the following equation: P_app_ = (V/(A x C_i_)) x (C_f_/T) where V is the volume of the basal chamber (mL), A is the area of epithelial layer (cm^2^), C_i_ is the initial concentration of the molecule (nM), C_f_ is the final concentration of the drug, and T is the assay time (s) [70]. The average P_app_ with a single standard deviation is shown (average ± SD). The efflux ratio was calculated according to the following equation: Efflux Ratio = P_app_(B-L)/P_app_(L-B) [16].

### Statistical analysis

Statistical analysis of ALP, Muc2, and Edu quantification as well as transporter proteomic analysis and the functional transport assays was performed as described above. For all permeability assays (LY, fluorescein-dextran, atenolol, propranolol, digoxin, prazosin), statistical analysis was performed with Tukey’s honest significant difference procedure conducted at the 5% significance level. In all figures ‘*’ denotes *p* < 0.05 and ‘**’ *p* < 0.005. The average with a single standard deviation (SD) is shown (average ± SD). All experiments were performed in triplicate unless specified otherwise.

## Additional file

Additional file 1: **Figure S1.** Day 5 Edu Staining of intestinal epithelial cells in expansion medium (EM). Fluorescence images of the monolayers at day 5 stained for EdU (green), and nuclei (blue). Scale bar = 100 μm. **Figure S2.** QTAP SRM quantification of selected transporters. Protein concentration (pmol/mg) of selected transporters of the monolayers in the thick collagen scaffold and thin layer collagen over the porous membrane at day 10 compared to that of fresh crypts/villi. **Figure S3.** Oxygen saturation evaluation. COMSOL Multiphysics simulations of oxygen saturation for (A) gradient cross-linked scaffold and (B) conventional scaffold. The oxygen saturation is shown 3 h after the start of a transport assay *i.e.* medium exchange. In the gradient cross-linked scaffold and conventional scaffold, the oxygen saturation in the luminal reservoir 1 mm above the cells was nearly identical for the two culture formats (10.1% and 9.5% for the gradient cross-linked and conventional formats, respectively). **Figure S4.** Karyotyping results. Cytogenetic analysis showing a normal human female karyotype at P15. **Table S1.** Detailed list of the differences between the gradient cross-linked scaffold system and conventional scaffold system. **Table S2**. Human transporter proteotypic heavy labeled tryptic peptides standards (purchased from Theracode JPT Inc., Acton, MA) and MRMs acquired. C-terminus R and K amino acids are ^13^C and ^15^N heavy labeled (shown in bold). The mass differences between labeled (shown) and unlabeled (not shown) R and K are 10 and 8 respectively. Mass shift for transition ions, between labeled and unlabeled, also depends on the charge state. The product ion for most heavy labeled peptide MRMs contains the heavy label. Peptide selection had been based on *in silico* assessment, crude peptide evaluation and available literature. Where necessary, peptides used when reporting concentrations are marked with **●**. (DOCX 368 kb)
