# Supplementary material for: Molecular transport through primary human small intestinal monolayers by culture on a collagen scaffold with a gradient of chemical cross-linking
Source: J Biol Eng. 2019 Apr 27;13:36. doi: 10.1186/s13036-019-0165-4 (PMC6487070; doi:10.1186/s13036-019-0165-4)
Supplement: Supplementary file 1 — Figure S1. Day 5 Edu Staining of intestinal epithelial cells in expansion medium (EM). Fluorescence images of the monolayers at day 5 stained for EdU (green), and nuclei (blue). Scale bar = 100 μm. Figure S2. QTAP SRM quantification of selected transporters. Protein concentration (pmol/mg) of selected transporters of the monolayers in the thick collagen scaffold and thin layer collagen over the porous membrane at day 10 compared to that of fresh crypts/villi. Figure S3. Oxygen saturation evaluation. COMSOL Multiphysics simulations of oxygen saturation for (A) gradient cross-linked scaffold and (B) conventional scaffold. The oxygen saturation is shown 3 h after the start of a transport assay i.e. medium exchange. In the gradient cross-linked scaffold and conventional scaffold, the oxygen saturation in the luminal reservoir 1 mm above the cells was nearly identical for the two culture formats (10.1% and 9.5% for the gradient cross-linked and conventional formats, respectively). Figure S4. Karyotyping results. Cytogenetic analysis showing a normal human female karyotype at P15. Table S1. Detailed list of the differences between the gradient cross-linked scaffold system and conventional scaffold system. Table S2. Human transporter proteotypic heavy labeled tryptic peptides standards (purchased from Theracode JPT Inc., Acton, MA) and MRMs acquired. C-terminus R and K amino acids are 13C and 15N heavy labeled (shown in bold). The mass differences between labeled (shown) and unlabeled (not shown) R and K are 10 and 8 respectively. Mass shift for transition ions, between labeled and unlabeled, also depends on the charge state. The product ion for most heavy labeled peptide MRMs contains the heavy label. Peptide selection had been based on in silico assessment, crude peptide evaluation and available literature. Where necessary, peptides used when reporting concentrations are marked with ●. (DOCX 368 kb) [file 13036_2019_165_MOESM1_ESM.docx]

**Additional file 1**

**Table of Contents**

01. Supplemental Methods

Comsol simulations of the oxygen concentration near the cells on the two platforms.

02. Supplemental Figures

Supplemental Figure S1. Day 5 Edu staining.

Supplemental Figure S2. QTAP SRM quantification of selected transporters.

Supplemental Figure S3. Karyotyping results.

Supplemental Figure S4. Modelling the oxygen saturation in the presence of cells on the two platforms.

03. Supplemental Tables

Supplemental Table 1. Detailed list of the differences between the gradient cross-linked scaffold system and conventional scaffold system.

Supplemental Table 2. Human transporter proteotypic heavy labeled tryptic peptides standards and (multiple reaction monitoring) MRM acquired.

04. Supplemental References

01. Supplemental Methods

**Comsol simulations of the oxygen concentration near the cells on the two platforms.**

COMSOL Multiphysics® was used to simulate the oxygen saturation at the luminal side of the cells for both culture systems. The diffusion coefficient of O_2_ through water was taken to be 3 ×10^-9^ m^2^ s^-1^ at 37 °C (1). The oxygen consumption rate (OCR) of the intestinal cells was modeled as -8.64 × 10^-3^ mol/m^3^ ∙ s (2). The model took into the difference in the height of the fluid above the cells.

02. Supplemental Figures

Figure S1.


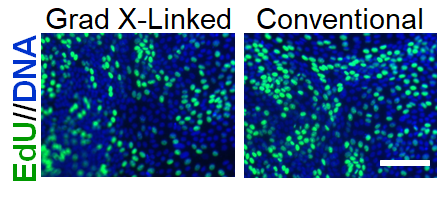


**Figure S1.** Day 5 Edu Staining of intestinal epithelial cells in expansion medium (EM). Fluorescence images of the monolayers at day 5 stained for EdU (green), and nuclei (blue). Scale bar = 100 µm.

Supplemental Figure S2.


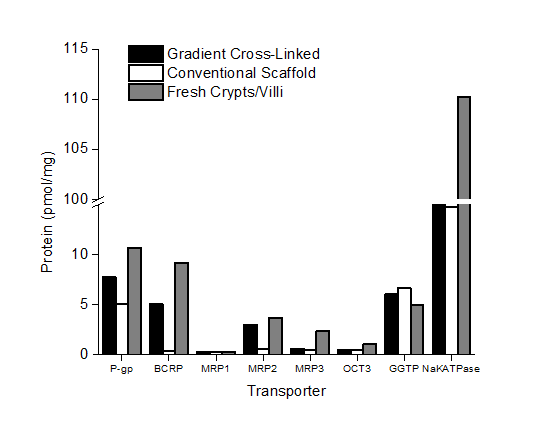
**Supplemental Figure S2.** QTAP SRM quantification of selected transporters. Protein concentration (pmol/mg) of selected transporters of the monolayers in the thick collagen scaffold and thin layer collagen over the porous membrane at day 10 compared to that of fresh crypts/villi.

Figure S3.


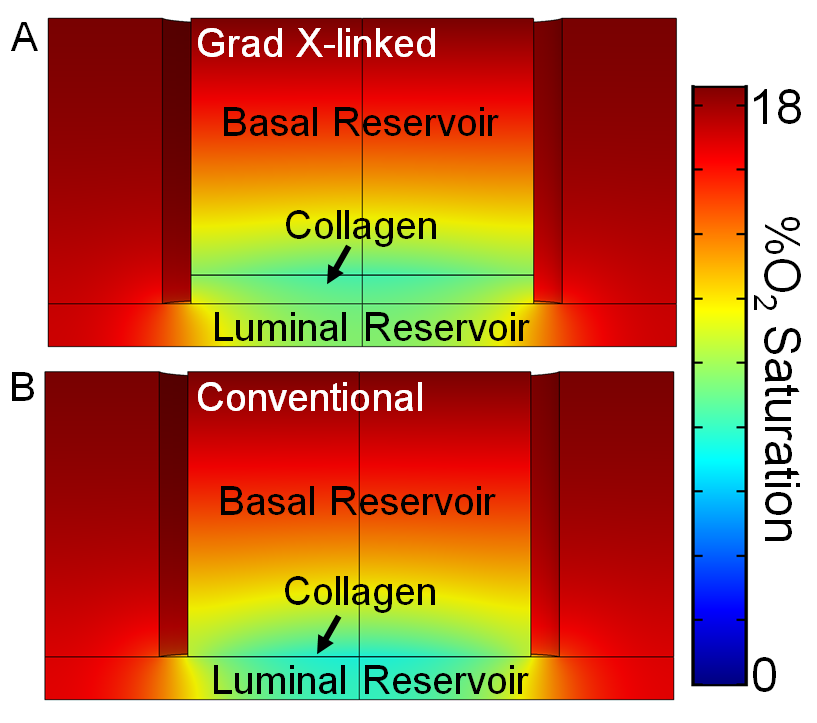


**Figure S3. Oxygen saturation evaluation.** COMSOL Multiphysics simulations of oxygen saturation for (A) gradient cross-linked scaffold and (B) conventional scaffold. The oxygen saturation is shown 3 h after the start of a transport assay *i.e.* medium exchange. In the gradient cross-linked scaffold and conventional scaffold, the oxygen saturation in the luminal reservoir 1 mm above the cells was nearly identical for the two culture formats (10.1% and 9.5% for the gradient cross-linked and conventional formats, respectively).


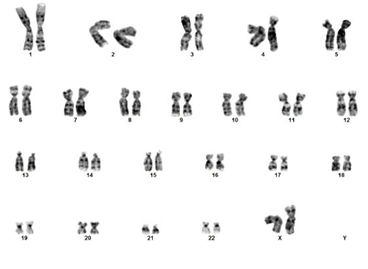
Figure S4.

**Figure S4.** Karyotyping results. Cytogenetic analysis showing a normal human female karyotype at P15.

03. Supplemental Tables

**Table S1.** Detailed list of the differences between the gradient cross-linked scaffold system and conventional scaffold system.

|  | **Gradient Cross-Linked Scaffold** | **Conventional Scaffold** |
| --- | --- | --- |
| **ECM Coating** | 1 mg/mL rat tail collagen | 0.1 mg/mL rat tail collagen + 0.01 mg/mL human collagen |
| **Collagen Thickness** | 1.2 mm | < 900 nm (rat + human collagen) |
| **Fluid Height Above the Cells** | 9 mm | 10 mm |
| **Stiffness** | 230 ± 140 Pa | 1.50 ± 0.27 MPa |
| **Membrane Material** | Polyethylene terephthalate (PET) | Polycarbonate (PC) |
| **Cell Growth Area** | 0.9 cm^2^ | 1.12 cm^2^ |
| **Nominal Pore Density** | 1.6 × 10^6^ pores/cm^2^ | 1.0 × 10^8^ pores/cm^2^ |
| **Membrane Pore Size** | 0.4 µm | 0.4 µm |
| **Membrane Diameter** | 10.5 mm | 12 mm |

**Table S2**. Human transporter proteotypic heavy labeled tryptic peptides standards (purchased from Theracode JPT Inc., Acton, MA) and MRMs acquired. C-terminus R and K amino acids are ^13^C and ^15^N heavy labeled (shown in bold). The mass differences between labeled (shown) and unlabeled (not shown) R and K are 10 and 8 respectively. Mass shift for transition ions, between labeled and unlabeled, also depends on the charge state. The product ion for most heavy labeled peptide MRMs contains the heavy label. Peptide selection had been based on *in silico* assessment, crude peptide evaluation and available literature. Where necessary, peptides used when reporting concentrations are marked with **●**.

| **Transporter (Gene)** | **Peptide Sequence** | **MRM 1**  **(product ion)^a^**  **(Mass Spec Specific)** | **MRM 2**  **(product ion)**  **(Mass Spec Specific)** |
| --- | --- | --- | --- |
| P-gp (MDR1, ABCB1) | F_1086_YDPLAG**K**_1093_ | 459.75/608.36 (y6) | 459.75/493.33 (y5) |
| P-gp (MDR1, ABCB1) | I_368_IDNKPSIDSYS**K**_380_^b^**●** | 496.60/631.32 (y11) | 496.60/904.46 (y8) |
| P-gp (MDR1, ABCB1) | N_809_TTGALTT**R**_817_ | 472.76/729.41 (y7) | 472.76/628.36 (y6) |
| P-gp (MDR1, ABCB1) | I_896_ATEAIENF**R**_905_ | 587.31/759.40 (y6) | 587.31/688.37 (y5) |
| BCRP (ABCG2) | V_164_IQELGLD**K**_172_ | 511.81/810.46 (y7) | 511.81/682.40 (y6) |
| BCRP (ABCG2) | L_332_AEIYVNSSFY**K**_343_ | 721.38/1015.51 (y8) | 721.38/852.44 (y7) |
| BCRP (ABCG2) | S_87_SLLDVLAA**R**_96_**●** | 527.81/654.38 (y6) | 527.81/767.47 (y7) |
| BCRP (ABCG2) | E_138_NLQFSAAL**R**_147_ | 579.81/674.39 (y6) | 579.81/802.45 (y7) |
| BCRP*2 (ABCG2*2) | F_142_SAAL**R_147_** | 337.70/440.29 (y4) | 337.70/527.32 (y5) |
| MRP1 (ABCC1) | T_1067_PSGNLVN**R**_1075_**●** | 484.26/433.74 (y8) | 484.26/769.42 (y7) |
| MRP1 (ABCC1) | A_1188_YYPSIVAN**R**_1197_ | 582.31/766.45 (y7) | 582.31/398.17 (b3) |
| MRP2 (ABCC2) | Y_514_FAWEPSF**R**_522_ | 606.79/902.44 (y7) | 606.79/516.28 (y4) |
| MRP2 (ABCC2) | Q_1059_LLNNIL**R**_1066_ | 497.31/752.47 (y6) | 497.31/639.38 (y5) |
| MRP2 (ABCC2) | V_803_LGPNGLL**K**_811_ | 459.80/706.44 (y7) | 459.80/819.53 (y8) |
| MRP2 (ABCC2) | L_1377_TIIPQDPILFSGSL**R**_1392_**●** | 890.52/441.31 (b4) | 890.52/999.59 (y9) |
| MRP3 (ABCC3) | A_161_EGEISDPF**R**_170_ | 565.77/631.31 (y5) | 565.77/429.25 (y3) |
| MRP3 (ABCC3) | G_654_ALVAVVGPVGCG**K**_667_**●** | 646.37/682.35 (y7) | 646.37/781.42 (y8) |
| MRP4 (ABCC4) | D_634_NEESEQPPVPGTPTL**R**_650_ | 625.97/376.22 (y7) | 625.97/751.43 (y7) |
| MRP4 (ABCC4) | E_1157_TIEDLPG**K**_1165_ | 505.27/666.37 (y6) | 505.27/537.32 (y5) |
| MRP4 (ABCC4) | E_1022_APWEYQ**K**_1029_ | 529.76/429.72 (y6) | 529.76/858.43 (y6) |
| MRP4 (ABCC4) | A_808_PVLFFD**R**_815_ | 487.77/707.38 (y5) | 487.77/452.25 (y7) |
| MRP5 (ABCC5) | S_456_LSEASVAVD**R**_466_ | 572.30/943.47 (y9) | 572.30/656.36 (y6) |
| MRP6 (ABCC6) | A_282_PETEPFL**R**_290_ | 535.28/499.76 (y8) | 535.28/542.33 (y4) |
| MRP6 (ABCC6) | T_1142_QAPFVAQNNA**R**_1153_ | 663.84/301.15 (b3) | 663.84/1026.54 (y9) |
| MRP9 (ABCC12) | E_559_NILFGE**K**_566_ | 479.27/488.27 (y4) | 479.27/601.35 (y5) |
| MRP9 (ABCC12) | T_1291_DTLVQNTI**K**_1300_ | 570.83/710.44 (y6) | 570.83/611.37 (y5) |
| ENT1 (SLC29A1) | D_59_AQASAAPAAPLPE**R**_73_ | 492.26/621.36 (y5) | 737.88/860.49 (y8) |
| ENT1 (SLC29A1) | W_360_LPSLVLA**R**_368_ | 532.83/765.49 (y7) | 532.83/383.25 (y7) |
| OATP1A2 (SLCO1A2) | E_273_GLETNADII**K**_283_ | 605.83/782.46 (y7) | 605.83/681.41 (y6) |
| OATP1A2 (SLCO1A2) | Y_599_IYLGLPAAL**R**_609_ | 630.37/707.44 (y7) | 630.37/983.59 (y9) |
| OATP1A2 (SLCO1A2) | I_591_YDSTTF**R**_598_ | 506.75/736.35 (y6) | 506.75/621.32 (y5) |
| OATP2A1 (SLCO2A1) | S_26_VFGNI**K**_32_ | 386.73/586.35 (y5) | 386.73/439.29 (y4) |
| OATP2A1 (SLCO2A1) | V_239_NTAAVNLVPGDP**R**_252_ | 716.89/551.28 (y5) | 478.26/551.28 (y5) |
| OATP1B1 (SLCO1B1) | N_321_VTGFFQSF**K**_330_ | 591.81/969.50 (y8) | 591.81/868.46 (y7) |
| OATP1B1 (SLCO1B1) | L_402_NTVGIA**K**_409_ | 412.27/596.40 (y6) | 412.27/710.44 (y7) |
| OATP1B3 (SLCO1B3) | L_402_SLVGIA**K**_409_ | 404.78/608.43 (y6) | 404.78/695.46 (y7) |
| OATP1B3 (SLCO1B3) | I_615_YNSVFFG**R**_623_ | 556.79/836.43 (y7) | 556.79/722.34 (y6) |
| OATP2B1 (SLCO2B1) | V_363_LLQTL**R**_369_ | 426.78/640.40 (y5) | 426.78/527.32 (y4) |
| OATP2B1 (SLCO2B1) | Y_641_YNNDLL**R**_648_ | 540.77/754.41 (y6) | 540.77/917.47 (y7) |
| OATP2B1 (SLCO2B1) | S_687_SPAVEQQLLVSGPG**K**_702_ | 802.95/715.91 (y14) | 535.63/552.33 (y6) |
| OATP4C1 (SLCO4C1) | L_201_GSLFEDTCVTT**R**_213_ | 754.87/991.44 (y8) | 754.87/1138.51 (y9) |
| OATP4C1 (SLCO4C1) | G_654_ACWIYDNI**K**_663_ | 624.31/660.35 (y5) | 624.31/773.44 (y6) |
| OAT3 (SLC22A8) | F_76_VHPPNASLPNDTQ**R**_90_ | 568.29/370.68 (y6) | 568.29/740.36 (y6) |
| OAT3 (SLC22A8) | T_412_VLAVFG**K**_419_ | 421.77/642.42 (y6) | 421.77/529.33 (y5) |
| OCT2 (SLC22A2) | S_313_LPASLQ**R**_320_ | 441.26/341.20 (y6) | 441.26/681.39 (y6) |
| OCT2 (SLC22A2) | S_53_PGVAELSL**R**_62_ | 519.79/698.41 (y6) | 519.79/476.28 (y9) |
| OCT3 (SLC22A3) | G_522_IALPETVDDVE**K**_534_**●** | 697.37/1039.51 (y9) | 697.37/355.23 (b4) |
| OCT3 (SLC22A3) | F_213_LQGVFG**K**_220_ | 452.27/643.38 (y6) | 452.27/515.32 (y5) |
| OCTN1 (SLC22A4) | A_327_FILDLF**R**_334_ | 502.79/673.39 (y5) | 502.79/786.48 (y6) |
| NaK ATPase (ATP1A1) | V_213_DNSSLTGESEPQT**R**_227_**●** | 543.92/511.29 (y4) | 815.38/511.29 (y4) |
| Gamma GTP (GGT1,2,3P) | L_156_FQPSIQLA**R**_165_**●** | 591.85/794.48 (y7) | 591.85/389.22 (b3) |

^a^ MRMs are roughly in order of highest intensity. ^b^ KP is a missed cleavage site.

04 References.

1. Buchwald P. FEM-based oxygen consumption and cell viability models for avascular pancreatic islets. Theor Biol Med Model. 2009;6(5).

2. Lin CS, Liu LT, Ou LH, Pan SC, Lin CI, Wei YH. Role of mitochondrial function in the invasiveness of human colon cancer cells. Oncol Rep. 2018;39(1):316–30.
